# A Supervised Machine Learning Approach to Classify Brain Morphology of Professional Visual Artists versus Non-Artists

**DOI:** 10.3390/s23094199

**Published:** 2023-04-22

**Authors:** Alessandro Grecucci, Clara Rastelli, Francesca Bacci, David Melcher, Nicola De Pisapia

**Affiliations:** 1 Department of Psychology and Cognitive Sciences of Trento, University of Trento, 38068 Rovereto, Italy; 2 MEG Center, University of Tübingen, 72072 Tübingen, Germany; 3 College of Arts and Creative Enterprises, Zayed University, Abu Dhabi P.O. Box 144534, United Arab Emirates; 4 Division of Science, New York University Abu Dhabi, Abu Dhabi P.O. Box 129188, United Arab Emirates

**Keywords:** magnetic resonance imaging (MRI), visual arts, creativity, supervised machine learning, artists, gray matter, multi-kernel learning, imagery

## Abstract

This study aimed to investigate whether there are structural differences in the brains of professional artists who received formal training in the visual arts and non-artists who did not have any formal training or professional experience in the visual arts, and whether these differences can be used to accurately classify individuals as being an artist or not. Previous research using functional MRI has suggested that general creativity involves a balance between the default mode network and the executive control network. However, it is not known whether there are structural differences between the brains of artists and non-artists. In this study, a machine learning method called Multi-Kernel Learning (MKL) was applied to gray matter images of 12 artists and 12 non-artists matched for age and gender. The results showed that the predictive model was able to correctly classify artists from non-artists with an accuracy of 79.17% (AUC 88%), and had the ability to predict new cases with an accuracy of 81.82%. The brain regions most important for this classification were the Heschl area, amygdala, cingulate, thalamus, and parts of the parietal and occipital lobes as well as the temporal pole. These regions may be related to the enhanced emotional and visuospatial abilities that professional artists possess compared to non-artists. Additionally, the reliability of this circuit was assessed using two different classifiers, which confirmed the findings. There was also a trend towards significance between the circuit and a measure of vividness of imagery, further supporting the idea that these brain regions may be related to the imagery abilities involved in the artistic process.

## 1. Introduction

Creativity is one of the cognitive processes that allows humans to make art, and it drives cultural and technological progress in our society [1,2,3,4,5]. In addition, creative ability has an important role in individual subjective well-being at any age [6,7,8] since it has therapeutic value [9] and is a predictor of academic success [10] as well as artistic prowess [11]. The artistic process functions as a cohesive communicative system. It conveys cultural, emotional, and aesthetic information, often through symbolic means which are culturally and context-specific [12]. Visual art, including drawings and sculptures, is a particularly important form of creative expression that has a long history dating back to (at least) the Upper Paleolithic period [13,14]. There is rich evidence of its practice across all cultures and, diachronically, throughout human history.

Because of these factors, visual art is a particularly interesting subject for the study of the cognitive and neural processes underlying creativity. The science of creative cognition has traditionally focused on the study of behavior, including convergent and divergent thinking [15,16]. Over the past few decades, the literature has branched out by shifting the focus to a more complex construct of creativity [17,18], accounting for the many different manifestations of creativity and the enormous variability in the capacity of being creative among individuals. Since then, an increasing number of studies have made considerable progress, applying neuroimaging techniques to explore how creative thinking is manifested in the function and structure of the brain [17,19,20], with a growing interest in studying specific domains such as the visual domain (e.g., [21,22,23]). However, creativity research has been identified as a critically poorly studied field in neuroscience [24], and the differences in human brain anatomy underlying visual artistic creativity have only scarcely been examined [25,26,27,28].

Most previous research on the neurocognitive processes of creativity has used functional magnetic resonance imaging (MRI) to observe brain networks and identify spatially distributed brain regions that show a specific pattern of activity during rest or creative tasks [29]. Several studies have consistently identified a pattern of functional connectivity between two major large-scale brain networks, the executive control (ECN) and default mode (DMN), during creative performance [23]. The ECN mainly encompasses lateral nodes of the dorsolateral prefrontal cortex (DLPFC) and posterior parietal regions [30]. The ECN activation has been found to mediate planning and abstract reasoning, including the capacity to enable the retrieval, retention, inhibition, integration, and evaluation of mental representations [31,32,33,34,35]. The ECN has been shown to play a significant role in visual generative processes [21,22,23]. In contrast, the DMN was found to be active in the absence of current external stimuli and linked to highly integrated and complex behavioral functions, including self-referential or spontaneous thought, such as mind-wandering, episodic and semantic memory, as well as visual–spatial information and visual imagery [36,37,38]. These processes, fundamental to visual creativity, are mainly mediated by regions within the medial prefrontal cortex (MPFC), the posterior cingulate cortex (PCC), the temporal lobes (TL), the precuneus, and the temporoparietal junction (TPJ) [38]. Recent studies have found that TL activates during drawing tasks [21,39]. Activation in the left anterior hippocampus of the TL has been linked to the formation of novel conceptual combinations [40]. A recent meta-analysis [41] found that drawing creativity unveiled a variety of significant activations related to visual imagery and motor control, including but not limited to the inferior parietal lobule (IPL), the superior occipital gyrus (SOG), and the middle and inferior frontal gyrus (IFG). Together, these findings indicate that regions such as the cingulate and parietal, occipital, and temporal regions within the DMN and ECN support visual creativity and imagery processes that are known to be shaped by expertise and training.

An interesting aspect of visual creative ability is the differences that exist between individuals, such as experts and non-experts. Creative individuals build on existing knowledge to form novel conceptual associations [42]. For instance, visual imagery, drawing skills, and abilities in fields such as science or sports can all be improved with expertise and training. This is because the brain has a strong capacity for plasticity, meaning it can constantly change its structure and function in response to the environment [43].

However, studying experts or individuals with training in art may not be the same as studying professional artists who have been recognized by critics and experts in their field. Professional artists may possess more developed and unique creative skills that may not be present to the same extent in other skilled or trained individuals. Our group previously conducted one study comparing the brains of professional artists, recruited in collaboration with a museum of contemporary art, to non-professional artists using functional MRI. The results showed that professional artists had enhanced connectivity between regions of the DMN and ECN compared to non-professionals [23].

However, despite functional differences, it is also interesting and important to consider whether the brain structure of artists is different from that of non-artists. There have been relatively few studies on the structural differences between professional artists and non-artists. Most research in this area has used functional MRI to examine the creative process in non-professional artists as a state-level variability. However, brain morphology is known to organize much of the functional activity observed with functional MRI [44]. Therefore, measuring brain structure, which is thought to underlie functional neural signals and behavior [45,46], is an appropriate way to investigate differences between individuals at the trait level. Such differences could reflect neural plasticity, in terms of expertise developed over years of practice, or also pre-existing neural predispositions towards certain artistic activities, or “talent” [47,48].

Previous studies have explored the relationship between visual artistic creativity and brain anatomy. Several studies have applied major emphasis on the study of brain injuries [14] and suggested that visual creativity is mainly mediated by the right lateral prefrontal cortex [49], the right neocortex [50], the left ventral thalamus [51], the bilateral frontal temporal lobe, the anterior hippocampus, the bilateral temporal pole, the inferior temporal gyrus, MTG, and the left amygdala [52]. This line of research has led to inconsistent findings, and advances in neuroimaging techniques have given new insights on structural patterns of the creative brain, measuring regional gray and white matter (e.g., [20,53,54,55]).

To date, only a few studies have examined how structural connectivity relates to visual creative capacities [25,26,27]. A recent study by Schlegel and colleagues [27] demonstrated a reorganization of prefrontal white matter in the artist compared to the control group. Another study has identified a positive relationship between artistic training and gray matter density in the cerebellum and medial frontal gyrus (MFG), and particularly in the right precuneus [25].

More recently, Shi and colleagues [26] compared artistic and scientific creativity, as assessed by the Creative Achievement Questionnaire, to gray volumetric matter (GMV) measures. The authors found that artistic creativity was negatively associated with GMV within a core region of the Salience Network, the anterior cingulate cortex (ACC) and SMA, whereas scientific creativity was positively associated with the ECN and semantic processing regions. Finally, a recent study used voxel-based morphometry (VBM) to identify different behavioral and brain mechanisms between art major students and non-art major students by using the figural Torrance Test of Creative Thinking. The authors found a correlation between figural scores and GMV of the left ACC and the left MFG [28]. These findings suggest that brain structure may play a role in our understanding of visual creative cognition and mental imagery. Although there have been a few morphometric studies conducted on this topic, they are relatively recent and limited in number, and a definitive conclusion has not yet been reached. Therefore, more extensive research is needed to gain a comprehensive understanding of the anatomical foundation of visual creativity.

While there are several mental abilities that can differentiate visual artists from non-artists (such as emotional sensitivity, visual memory, motor abilities, etc.), these aspects are beyond the scope of this study; rather, the specific purpose of this study is to determine whether there are structural differences in the brains of professional artists compared to non-artists, and whether these differences can be used to classify individuals as artists or non-artists. To the best of our knowledge, no previous study has examined these structural differences between professional artists and non-artists. Despite some progress in this field [25,26,27,28], studies exploring whether differences in brain morphology characterize visual artistic creativity and mental imagery are lacking. In the present study, we capitalized on the previous study [23] by analyzing the same participants but focusing this time on the structural properties of the brain, e.g., gray matter (GM) volume.

In addition to univariate approaches, such as voxel-based morphometry, machine learning techniques, also known as multi-voxel pattern analysis (MVPA) in the context of neuroscience, can be used to reveal important insights into brain activity and structure. MVPA is inherently multivariate, taking into account information from multiple voxels and being sensitive to spatially distributed effects [56], thus allowing a better categorization of whole-brain distributed networks. Importantly, supervised machine learning (SML) can be used to predict continuous or categorical data, for example, separating patients from healthy individuals [57,58,59,60], or specific categories of individuals, thus enabling the discovery of specific functional or structural features that separate one category from the other. As such, SML techniques build high-dimensional classifiers based on multivariate methods that assess multiple voxels within the brain space [59,61].

One algorithm used in supervised machine learning is Multiple Kernel Learning (MKL), which is a sparse machine learning method that identifies the most relevant features for classification. MKL is a form of Support Vector Machine using kernels to implement a priori anatomical knowledge, reduce computational complexity, and avoid overfitting. As such, MKL can make predictions based on anatomical localization and determine the most important brain regions contributing to group classification through a hierarchical model that estimates the weight contribution of each region to the model [62]. This algorithm allows us to estimate the contribution of each area in a whole-brain approach. The estimation follows a hierarchical organization from the most important region contributing to the model (in terms of explained variance) to the least important regions. By considering the most important regions, a “brain circuit” that mostly enables the statistical model to correctly classify and predict the groups (labels) of interest can be outlined. Another advantage of supervised machine learning models is that their ability to generalize to the general population is not assumed, but rather empirically tested through predictive accuracy. This means that these models can be used to predict new, unobserved cases.

We expect that professional visual artists will have developed more efficient brain function in areas involved in planning and creating artworks, since they are trained for years in complex tasks entailing decision making such as selecting, evaluating for visual saliency, comparing, rendering, and simplifying. Nine out of twelve participants, in fact, were trained in formal artistic education (art school and/or academy), which is a three- to four-year course of study teaching the fundamentals of visual elements such as color, form, line, shape, space, texture, and value. Typically, students have to master sketching, drawing, and painting from real models, understanding and rendering proportions, color, and tone and mastering pencil control, brush strokes, modeling, building, and shaping three-dimensionally in different materials. Self-taught artists practice the same skills, although in their own time and through a less structured approach.

Therefore, the areas that we focused on are the parietal regions, and areas related to visual and imagery abilities such as the occipital lobe and thalamus, as well as semantic areas such as the temporal lobe. These areas are also included in the ECN and DMN. This would provide further evidence for the role of these networks in the creative process as demonstrated by professional artists. In addition, we predicted that professional artists would show brain structure differences in emotion-related areas such as the amygdala, basal ganglia, and cingulate. Artistic products are often thought to convey emotions in the viewer [63], and we expected that professional artists would have structural changes in these areas due to their heightened emotional sensitivity.

To further investigate the relationship between the brain circuits that distinguish professional from non-professional artists, we included a measure of the vividness of imagery in our analysis. Our choice of measure was purely explorative, and it was informed by studies which found significant relationships between image vividness and visual art involvement, as well as spatial abilities and mental synthesis tasks [64,65]. We used the Vividness of Visual Imagery Questionnaire (VVIQ-2) to assess the vividness of imagery in both professional artists and control participants. We expected to find a positive correlation between the imagery measures and brain structure, based on previous results using different tasks and measures.

## 2. Materials and Methods

### 2.1. Participants

This study included a sample of 24 participants. The experimental group consisted of 12 professional artists who were recruited with the assistance of the Museum of Modern and Contemporary Art (MART) in Rovereto, Italy. The control group consisted of 12 non-artists who were not involved in artistic activities and were recruited through local advertisements. To ensure that the control participants did not have any involvement in visual artistic activities, we asked them before the study if they were professional artists or had any involvement in visual arts, and they confirmed that they did not. Additionally, in the previous functional study with these same participants [23], all participants were required to make a drawing after the MRI session. This allowed us to verify that none of the control participants had any graphical or drawing ability at a professional level. The two groups were matched for age (mean age of artists = 30.9, mean age of non-artists = 29.76, *p* = 0.58) and gender (4 female artists, 8 male artists; 6 female non-artists, 6 male non-artists, *p* = 0.43). All participants were right-handed and had no history of psychiatric or neurological disorders. This study was approved by the University of Trento Ethics Committee and all participants provided informed consent. Moreover, the participants consented to be included in a museum exhibition detailing the anonymized experiment findings and the resulting sketches and artworks (In Resonance: Snapshots of Creativity in the Brain. MART, Rovereto, Italy) [66]. They received reimbursement for travel expenses. As a further incentive, their work was featured in the forenamed exhibition and its illustrated catalogue.

### 2.2. Imagery Questionnaire

All participants were screened using the Vividness of Visual Imagery Questionnaire (41), a self-assessment of visual imagery ability. Participants were asked to rate their experience of visual imagery on 16 questions, ranging on a scale from 1 to 5, where 1 signified “Perfectly clear and as vivid as normal vision” and 5 represented “No image at all (only “knowing” that you are thinking of the object)”.

### 2.3. Brain Data Collection

Participants were screened for MRI compatibility by a medical doctor, and if suitable, took part in the MRI scanning session. The scan consisted of both anatomical and functional imaging data. Anatomical data were collected for the first 6 min of the session, followed by functional imaging in the experimental session (please refer to [23]). Brain data were acquired using a 4 T Bruker MedSpec Biospin MR scanner and a birdcage transmit, 8-channel radio-frequency head receiver coil. Head motion was restricted using foam padding surrounding the head. T1-weighted anatomical scans (MP-RAGE; 1 × 1 × 1 mm^3^; FOV, 256 × 224 mm^2^; 176 slices; GRAPPA acquisition with an acceleration factor of 2; TR, 2700 ms; TE, 4.18 ms; inversion time (TI), 1020 ms; 7° flip angle) were acquired.

### 2.4. Preprocessing

The T1-weighted images were pre-processed through SPM12 (Statistical Parametric Mapping, https://www.fil.ion.ucl.ac.uk/) [67] and CAT12 toolbox (Computational Anatomy Toolbox for SPM, http://www.neuro.uni-jena.de/cat/) [68]. First, all the images were re-oriented by placing the anterior commissure as the origin. Then, the segmentation into gray matter, white matter, and cerebrospinal fluid was performed. The registration was computed through Diffeomorphic Anatomical Registration using Exponential Lie algebra tools for SPM12 (DARTEL) [69]. Finally, the normalization to the MNI space with a spatial Gaussian smoothing of 10 was performed.

### 2.5. Supervised Machine Learning Procedure

The voxels belonging to the gray matter (GM) images of every subject were entered into a Multi-Kernel Learning classification model (MKL), a form of Support Vector Machine that relies on kernels to reduce computational complexity and reduce the possibility of overfitting, as implemented in PRoNTo toolbox (version 2.1, http://www.mlnl.cs.ucl.ac.uk/pronto). To allow the classifier to learn the predictive function that separates the groups, two categories were created: artists and non-artists (first step). Then, the GM images were entered and paired with the correct labels. In a second step (feature extraction), voxel-based values representing local GM volume were extracted from raw brain scans and aligned as vectors and then transformed into similarity matrices (kernels) to avoid the “curse of dimensionality problem”, allow a separation between classes, and simplify calculations [62]. MKL generates kernel matrices by using the GM voxels of every region (as specified by the anatomical atlas selected). In other words, the kernels incorporate anatomical a priori knowledge (not one general kernel matrix for the whole brain, but 116 kernel matrices, one for every anatomically specified brain region). This allows the system to compute the contribution of every region to the statistical model in the end. Kernel-based methods in machine learning have been shown to be valid methods enabling high predictive accuracy [70], even in the case of relatively small samples [71]. In the third step, the MKL was trained to associate the brains to their label categories to define a decision boundary. MKL has been shown to enhance the interpretability of the decision function and improve performance. Whole-brain analyses were performed using a general mask provided inside PRoNTo. Data were mean-centered and normalized, and age and gender were regressed out to avoid these potential confounds. The predictive function was defined during a training phase where the algorithm learned patterns from the provided data to predict a label (artist vs. non-artist). During a test phase, the algorithm was used to predict outcome in an independent dataset. Given the small sample size, we decided to use the Leave-One-Subject-Out (LOSO) cross-validation (instead of the k-folds method) to allow for generalization to new cases. The dataset was partitioned into “training” (all subjects minus 1) and “test” sets (the remaining subject). During the training phase, the MKL learned mapping between brain features and labels (artist vs. non-artist) on the training set. During the test phase, the learned function tried to predict the labels from the test set [72]. This step was repeated 24 times so that every subject was used as a test. The performance of the MKL was obtained by averaging the results from all the subfolds. Total accuracy, balanced accuracy, and predictive accuracy were considered to assess the classifier performance, as well as the area under the curve (AUC) of the receiver operator curve. The AUC is a summary measure of classifier performance, where higher is better (1 represents perfect performance, 0.5 represents random performance). In PRoNTo, the ROC and AUC are estimated within each iteration and then averaged across all the iterations. AUC is computed by using the proportion of true positive rates (TPR) against the false positive rates (FPR).

Hyperparameters were optimized as suggested by PRONTO developers with soft-margin C spanning 0.0001, 0.01, 1, 10, 100, and 1000 to compute the inner loop and the outer loop (model performance). PRONTO distinguishes two loops in the cross-validation scheme. The inner loop is used to train and test the model with each value of the hyperparameter specified by the user. The parameter leading to the highest performance in the inner loop (balanced accuracy for classification problems) is then used in the outer loop. For each fold of the outer loop, the model is trained using the “optimal” value of the hyperparameter and tested on the data that were left out.

Statistical significance of the classifications was estimated using 200 permutations with random assignment of group class to input image. For each iteration, the classification labels were randomly permuted across all participants and the cross-validation procedure was repeated. In the fourth step, the contribution of every brain region to the statistical model was computed. Of note, MKL is a whole-brain hierarchical model, meaning that the statistical contribution of every region (segregated by the atlas) is estimated from the best to the worst, i.e., regions are ordered according to the proportion of explained variance of the effect, from largest to smallest. However, to provide insight on the main regions leading to a correct classification, we consider only the regions with a percentage of contribution to the model equal or superior to 1%. Surf Ice software was used to plot the brain maps (https://www.nitrc.org/projects/surfice/).

## 3. Results

**Figure 1:**
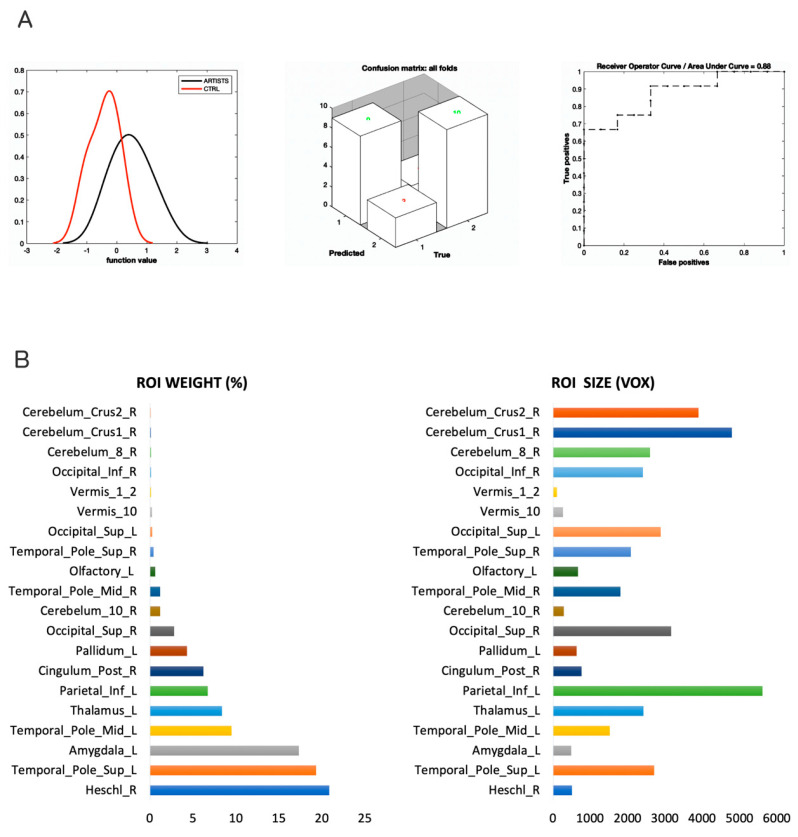
Performance of the MKL classifier and main regions contributing to the model. Multiple Kernel Learning machine classification based on structural (GM) features. (**A**) Left, density version of histogram plot of function values; center, confusion matrix; right, receiver operator curve, areas under the curve = 0.88. ROI weights in percentage and in voxel size are displayed in the two bar plots. (**B**) Bar plots with ROI weights (**left**) and voxel size (**right**) of the most predictive regions.

**Figure 2:**
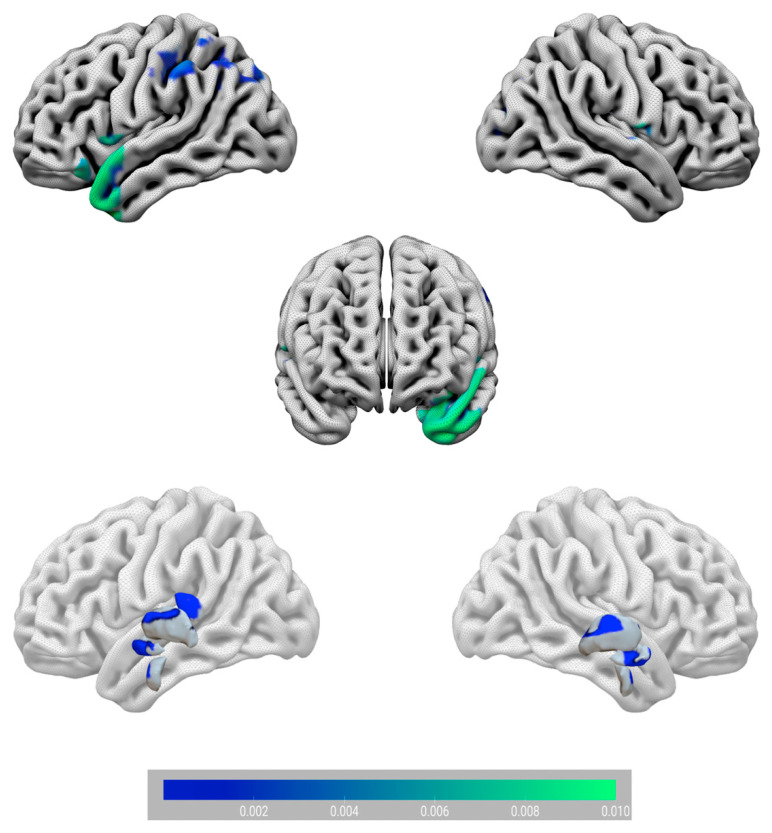
Surface plot of the circuity that correctly classified artists vs. non-artists. At a cortical level (**upper** part), the Heschl area, the temporal pole, the superior occipital, and parietal regions were the main contributors to the predictive model. At a subcortical level (**lower** part), the amygdala, the cingulate, the pallidum, and the thalamus were the most predictive regions. Note that cerebellar regions are not displayed as their weight contribution was below 1%. The bar plot indicates ROI weight values.

The MKL returned a total and balanced accuracy of 79.17% (*p* = 0.024), with a class accuracy of 75% for artists and 83.33% for non-artists. The class predictive value was of 81.82% for artists and 76.92% for non-artists, and the AUC was of 88% (Figure 1A). The model performance significantly exceeded the threshold of randomly guessing the labels, thus confirming that the algorithm successfully learned a predictive function [73] that can be used to predict new unobserved cases. Although MKL is a whole-brain estimation of the contribution of each of the 116 regions included in the atlas, we focus on the regions explaining at least 1% of the variance. GM regions were identified according to the Automated Anatomical Labeling (AAL) atlas (available on the WFU-PickUp Atlas toolbox of SPM12, [74]), a manual macroanatomical parcellation of single-subject MNI-template brain consisting of 116 brain regions. Regions with a larger contribution to the model were in the following order of importance: the right Heschl area, the left superior temporal pole, the left amygdala, the left mid-temporal pole, the left thalamus, the left inferior parietal, the right posterior cingulate, the left pallidum, and the right superior occipital areas. Cerebellar regions were also present but played a minimal role (below 1%). See Figure 1 and Figure 2.

**Figure 3:**
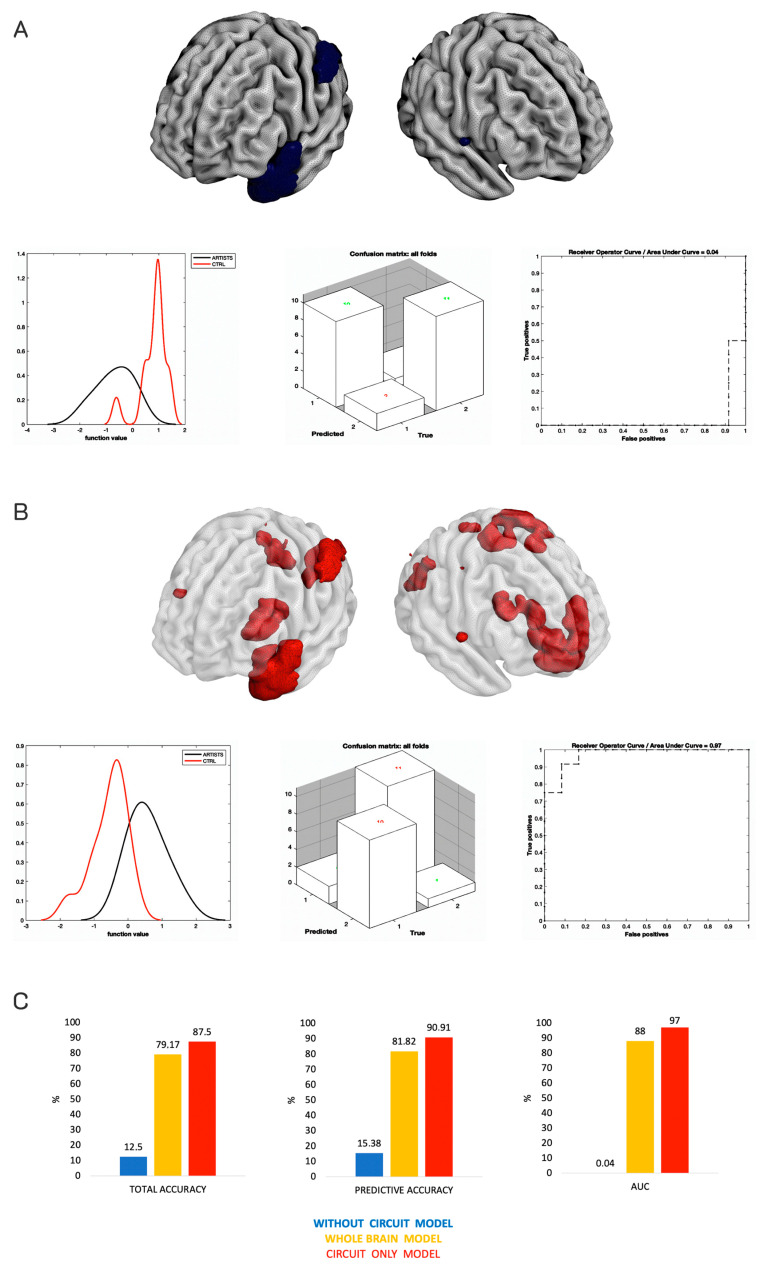
Testing the relevance of the MKL classifier circuit. (**A**) Drop in performance of the MKL classifier considering the brain features (in gray) minus the brain features of the predictive circuit of the main analysis (in dark blue). The classifier was unable to predict the classes and mislabeled the two groups. (**B**) Increased performance of the MKL classifier trained only on the brain features of the predictive regions of the main analysis (in red over the transparent brain). This classifier outperformed the original analysis by removing the probable noise provided by the non-relevant brain regions. (**C**) A comparison of the three models for total accuracy, predictive accuracy, and AUC.

To provide additional evidence that the circuit found in the MKL classifier was truly predicting being an artist, we re-ran the MKL classifier, this time removing the brain features belonging to that circuit. To execute this, in the feature set, we included all the voxels but the ones included in the circuit (via an ad hoc mask). This method may resemble the virtual lesioning method used in artificial neural networks to assess the supposed relevance of one module of the network in performing a given task. The performance was computed before and after the lesion of the module and the differences were considered. As expected, the performance of the MKL lesioned model dropped, with a total and balanced accuracy of 12.50% and class predictive values of 15.38% for artists and 9.09% for non-artists. The AUC was only 0.04%, demonstrating poor performance. Of note, the performance was below the random guessing (50% of accuracy). This means that the model was misclassifying the artists as non-artists, and the non-artists as artists. The classifier without these areas was not able to predict being an artist, and indeed mislabeled the two groups. Because of the poor performance of the lesioned model, permutations were not computed (see Figure 3A). Additionally, and for completeness, we ran another MKL classifier, this time using only the brain features belonging to the predictive circuit found in the first analysis (included regions with weight ≥1%). As expected, the performance of the model increased compared to the whole-brain results (main ML model) with a total and balanced accuracy of 87.50% and class predictive values of 90.91% for artists and 84.62% for non-artists. The AUC was 97%. Permutations returned a *p*-value of 0.005. See Figure 3B,C for a comparison of all models.

### Additional Analyses

**Figure 4:**
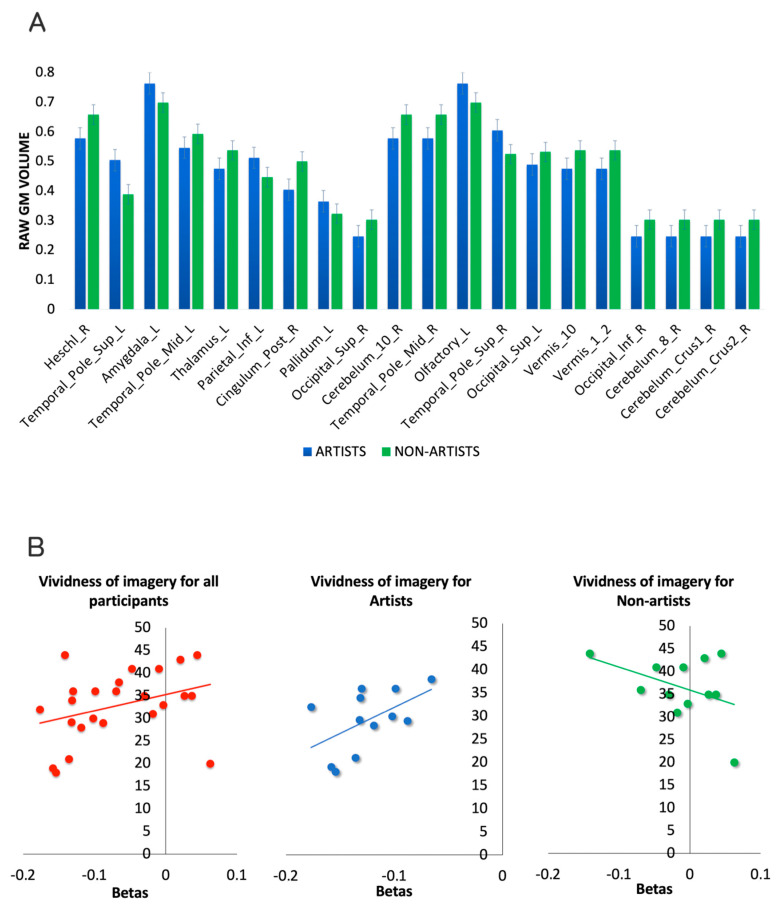
Additional analyses. (**A**) VBM was used to extract the raw GM volume of the regions of the MKL circuit. (**B**) Correlations between betas of the MKL classifier and of the vividness of imagery questionnaire for the whole group (**left**) and separated for artists (**central**) and non-artists (**right**).

To better characterize the direction of the effects in the GM volume of the regions found to be predictive of being an artist in the MKL classifier, we also computed voxel-based morphometry (VBM) limited to the circuit of interest (masked with MKL model of the main analysis). Total intracranial volume (TIV) was used as a control factor. Raw GM volumes were then extracted region by region via simple contrast with a threshold of *p* < 0.001. Among others, the temporal and the parietal regions as well as the amygdala and pallidum had increased raw GM volume in artists. The other regions displayed the opposite pattern. The interested reader can appreciate the direction of all the areas by visually inspecting the bar plot of Figure 4A.

To assess eventual relations between the betas of the circuit that separate artists from non-artists and the vividness of imagery questionnaire, we computed a non-parametric Spearman correlation. Of note, the VOI significantly differed between groups (t = 2.642, *p* = 0.015). The results showed a positive trend toward significance (rho = 0.376, *p* = 0.07), partially confirming a relation between the circuit that predicts being an artist and the ability of imagery. Of note, when splitting the two groups, artists again displayed a positive trend toward significance (rho = 0.542, *p* = 0.085), whereas for non-artists, we found a non-significant negative correlation (rho = −0.286, *p* = 0.367), further indicating a possible specific relationship between the predictive circuit and vividness of imagery specific for professional artists (see Figure 3B). In the same vein, we computed a correlation between vividness of imagery and function values of the main MKL model, indicating how distant every participant is from the hyperplane separating the two groups (larger values indicate larger brain differences from the point at which no differences can be detected between the two groups). This correlation returned a trend toward significance (rho = −0.401, *p* = 0.052). 

## 4. Discussion

In this study, we found that the brain regions most important for classifying visual artists and non-artists were the Heschl area, amygdala, cingulate, thalamus, and portions of the parietal, occipital, and temporal lobes. Additional analysis showed that this brain circuit is necessary for accurate classification and may be related, at least in part, to imagery abilities. To find these results, we used the MKL classifier, a type of supervised machine learning, which produced a model that was able to discriminate between the two groups with an accuracy of 79.17% (meaning the model correctly guessed the label 8 out of 10 times) and an area under the curve (AUC) of 88%, which is considered a “very good” performance. The model also had an accuracy of 81.82% in predicting whether an individual was a professional artist, indicating that it can be used to classify new cases. In the following sections, we discuss these findings in more detail.

Prior to this research, functional and structural studies have discovered that highly creative individuals share alterations within temporal areas related to the DMN and parietal regions included in the ECN (e.g., [19,21,55,73]), especially in prominent artists as compared to novices [23]. For what concerns the regions usually attributed to the DMN, we found numerous regions that overlapped with the DMN as discriminative between the two samples, mostly within the temporal lobe (bilateral STL and MTL), and the right PCC. These results are in contrast to those of Shi et al. [26], which found that the temporal lobe was not significantly correlated with artistic creativity. Indeed, a growing number of studies have revealed that the temporal lobe is closely linked to creative cognition and creative insight [20,75,76]. These cortical regions are thought to be in charge of episodic and semantic memory and are suggested to play a major role in the generation of original ideas [77] and mental representation [75]. Particularly, previous studies found that TL was involved when unrelated concepts were linked together or during a changing of representation [78]. The STL has been shown to be active during imagination tasks [79,80]. Another recent study looking at creative artistic potential found decreased STL volume [55]. The authors interpreted this finding in light of the hypothesis that the more proficiently one acquires a specific ability, cortical thinning reflects greater efficiency and lower degrees of plasticity [76]. Along with STL, evidence of the implication of MTL comes from numerous studies that have shown its activation during creative drawing tasks (e.g., [21,39]). Given its central role in episodic and declarative memory, engagement of the MLT may represent the generation of old ideas (e.g., [81]). Finally, PCC activation found consistent evidence within functional studies on creativity (e.g., [23,82]), and altered PCC was found when looking at creative achievements [76,83].

For what concerns the role of regions usually included in the ECN, we only found partial evidence of involvement of the ECN anatomical regions in separating artists from non-artists. Indeed, we found the posterior portion of the ECN to be involved, namely the left inferior parietal area. This region has been associated with novel idea generation [84,85], as well as mental simulation, imagery, and future thought, and was found to facilitate the discrimination of novel and familiar information in retrieval studies.

We also observed alteration of GMV distinguishing artists vs. non-artists in the left amygdala. This finding is consistent with other studies which demonstrate the involvement of the amygdala in artistic creativity, highlighting its role in spontaneous and emotional functioning [86]. A recent study found increased left-hemisphere amygdala volume to correlate with musical creativity [87]. However, another work found higher artistic creativity in relation to decreased left amygdala volume [55]. In addition, the GMV alteration of bilateral SOG, right cerebellum, left pallidum, and left thalamus are perhaps not surprising given that the region’s role has been largely associated with visual imagery and motor control. Overall, these brain regions support various higher-order cognitive functions such as visual and motor imagery, along with the processing and integration of sensorimotor and somatosensory information [88,89].

Interestingly, the right Heschl’s gyrus was discovered to be the most important cluster in distinguishing the samples. This result is not new if we consider studies looking at creativity [90], mainly within the music domain (e.g., [86,91]), perhaps because it represents the first structures to process incoming auditory and linguistic information. One possibility is that auditory processing or imagery may aid in creative thought. Alternatively, visual imagery might suppress auditory processing [92]. Interestingly, other studies have focused on the role of Heschl’s gyrus in relation to auditory verbal hallucinations in schizophrenia [93].

In addition, visual mental imagery abilities were found to be associated with occipital–temporal sensory regions of the brain. Precisely, we found a correlation approaching significance between mental imagery scores and the circuit. Rather than mere representations from specific episodic memories, some experiments on conscious manipulation have revealed that various areas collaborate as a network to create and deconstruct abstract mental images [94]. Among others, the superior occipital lobe may play a necessary role in basic visual perception (such as color/shape recognition) and visual imagery [41,75]. Its involvement has already been found in numerous studies on creative insight and insight-based problem solving [75]. Moreover, individuals engaged in visual imagery tasks seem to exhibit greater recruitment of the occipital lobe [95,96]. Another research compared experts and novices during a drawing task, showing activated occipital lobe in experts [30,97]. As such, our findings indicate that the FG supports visual creativity processes that are known to be shaped by expertise and training.

Our study does not come without limitations, including a small sample size of artists and controls. The former were difficult to recruit due to the requirements for professional recognition. It is worth noting that the generalizability of predictive models with limited sample sizes has been called into question in recent research [98]. However, there is also evidence that a bigger sample size does not improve bias in performance estimates when this validation method is used [59,61,99,100]. Further research with larger sample sizes is needed to confirm our findings. Additionally, we only observed a trend towards significance when using a questionnaire to measure the vividness of imagery, which we believe may be due to the small sample size or the fact that the brain circuit being analyzed is involved in more than just imagery and creativity, such as emotion. Future studies may want to examine specific components of this circuit in more detail.

Another limitation of our study is related to the recruitment of participants. We only contacted visual artists through a museum of contemporary art, which may have led to a sample that is biased towards a particular type of artist. Additionally, the control group was recruited based on their lack of involvement in visual arts, but with no specific criteria regarding their profession or creative background. Future research could aim to recruit control groups with more specific characteristics, such as creatives in other artistic fields (e.g., music) or individuals from non-artistic professions (e.g., science), in order to better compare the cognitive abilities of visual artists with those of other groups.

One more limitation refers to the fact that to investigate the relationship between the brain circuits that distinguish professional from non-professional artists, we only examined the correlation between brain features and a measure of the vividness of imagery. While this aspect of cognition is a feature that might be linked to creativity (e.g., [101]), in future studies it would be interesting to make use of other ratings that might differentiate professional artists and control groups such as visuospatial and motor skills, visual memory, attention to detail, and the ability to mentally manipulate visual information (e.g., [101]).

Moreover, our study is limited by its cross-sectional design, which does not allow us to infer causality, and thus, we cannot conclude that the identified differences in brain circuits between artists and non-artists are solely due to brain plasticity. While previous studies suggest that brain plasticity may play a role in the development of artistic expertise, our study cannot prove this. To confirm the role of brain plasticity in the observed structural differences, future longitudinal studies are needed. Such studies could follow students of visual arts over a period of time to examine changes in brain structure as they progress in their training.

A further constraint of our research is that we focused on the structural side of the brain, ignoring other sources of data. A previous study from the authors of the present study [23] focused on the functional side of these subjects (resting state connectivity), but ignored the structural side. One possibility is that in a future study, both modalities could be combined in a data fusion approach to possibly maximize the accuracy of the model.

Our study is additionally limited by the use of MKL, a hierarchical whole-brain estimation method. This means that although we focus our attention on the regions contributing the most (e.g., regions with at least 1% of explained variance), other regions may be important in the accurate classification of being an artist or not. Future studies may want to explore this issue by implementing shrinkage methods to maximize the most relevant regions and minimize the role of the least important regions. Despite these limitations, this study represents a valuable initial effort to understand the brain differences between professional artists and non-artists, and provides new insights into the gray matter differences between these groups. Finally, the results from the second and third analyses should be interpreted with caution. Although the “circuit only model” confirms the goodness of the circuit derived from the first analysis (“whole brain model”), and the interpretation is quite straightforward (improved performance when considering the most important regions in the main analysis), the results from the “without circuit model” should be interpreted with more caution. The fact that the performance of the model worsens makes sense since the regions used in this model only accounted for less than 2% of the effect of the main analysis. However, the fact that the model misclassified artists as non-artists, and vice versa, is not so easy to interpret.

To conclude our discussion, we emphasize that our study employed a supervised machine learning (SML) method known as Multi-Kernel Learning to study the neural bases of professional artists vs. non-artists. SML methods have several advantages compared to standard univariate methods such as voxel-based morphometry or surface-based morphometry used in neuroscience. First, this method is a multivariate statistical approach. This means that this method takes into account the relationship between voxels and does not consider them independently. Voxels are just an arbitrary partitioning of the brain tissue not biologically driven. Considering them as independent units leads to even less biologically plausible parcellation of the brain. Furthermore, by considering the dependency among voxels, SML methods outperform univariate methods for their sensitivity in finding even subtle distributed neural differences between groups [59,61]. This is also more coherent with a network perspective in neuroscience for which psychological differences between groups cannot be ascribed to single regions but to larger networks of regions. Moreover, SML methods do not assume generalization as in standard frequentist approaches but provide an empirical estimation of generalization (usually reported in classification tasks as the percentage of accuracy, ROC/AUC, and other measures). The standard frequentist approaches (such as the General Linear Model used in univariate statistical approaches) assume that the results derived from the analyzed sample can be safely approximated to the general population by simply adding an error value. In other words, generalization is assumed and not tested. SML methods have been specifically designed to test for their generalization. This means that the results from our study can be used to predict new artists and non-artists by simply analyzing their brains with our model. Of all the SML methods, in the present study, we used a form of Support Vector Machine known as MKL that has the further advantage of taking into consideration anatomical information (atlas-based brain regions) to determine the most important regions contributing to the classification [62]. This allows an inference on the most important brain regions, allowing a correct classification of professional artists vs. non-artists. Thanks to this approach, we were able to understand the brain network that not only differs (in terms of GM) between the two groups, but that also allows a correct classification of professional artists vs. non-artists.

## 5. Conclusions

In conclusion, this study is the first to provide neural evidence of anatomical differences between the brains of professional artists and non-artists. We found differences in cortical and subcortical regions known to be related to creativity, visual and imagery abilities, and emotions. As a final consideration, we would like to mention the potential ethical implications of our research. Our aim with this study is to contribute to the broader understanding of the neural basis of creativity and artistic abilities, with potential applications in fields such education or possibly art therapy. Although such information can be beneficial in various domains, it is crucial to acknowledge the potential risks associated with employing classifier methods in a discriminatory manner or to infringe upon individual privacy. As for other areas of research in neuroscience (e.g., [102]), to ensure responsible and ethical conduct in the use of classifier methods, it is crucial for both scientists and society to engage in discussions about their ethical implications. It is recommended that guidelines and policies be established to regulate the use of these methods, and that educational and awareness campaigns be implemented to inform policy makers and the general public about the potential benefits and risks associated with the use of neuroscientific methods for classification purposes.

## Data Availability

Data are unavailable due to privacy and ethical restrictions included in the approval from the Ethics Committee of the University of Trento.

## References

[1] De Pisapia N., Rastelli C. (2022). Creativity as an Information-Based Process. Riv. Internazionale Filos. Psicol..

[2] Simon H.A. (1977). The Logic of Heuristic Decision Making. Models of Discovery.

[3] Feist G.J. (2006). The Psychology of Science and the Origins of the Scientific Mind.

[4] Catmull E., Wallace A. (2015). Srilakshmi Creativity Inc.: Overcoming the Unseen Forces That Stand in the Way of True Inspiration.

[5] Kaufman J.C., Sternberg R.J. (2010). The Cambridge Handbook of Creativity.

[6] Rastelli C., Greco A., De Pisapia N., Finocchiaro C. (2022). Balancing novelty and appropriateness leads to creative associations in children. PNAS Nexus.

[7] Dolan P., Metcalfe R. (2012). The relationship between innovation and subjective wellbeing. Res. Policy.

[8] Palmiero M., Nori R., Piccardi L. (2017). Verbal and visual divergent thinking in aging. Exp. Brain Res..

[9] Schouten K.A., de Niet G.J., Knipscheer J.W., Kleber R.J., Hutschemaekers G.J.M. (2014). The Effectiveness of Art Therapy in the Treatment of Traumatized Adults: A Systematic Review on Art Therapy and Trauma. Trauma Violence Abus..

[10] Gajda A., Karwowski M., Beghetto R.A. (2017). Creativity and Academic Achievement: A Meta-Analysis. J. Educ. Psychol..

[11] Carson S.H., Peterson J.B., Higgins D.M. (2005). Reliability, Validity, and Factor Structure of the Creative Achievement Questionnaire. Creat. Res. J..

[12] Zaidel D.W., Nadal M., Flexas A., Munar E. (2013). An Evolutionary Approach to Art and Aesthetic Experience. Psychol. Aesthet. Creat. Arts.

[13] Aubert M., Brumm A., Ramli M., Sutikna T., Saptomo E.W., Hakim B., Morwood M.J., Van Den Bergh G.D., Kinsley L., Dosseto A. (2014). Pleistocene Cave Art from Sulawesi, Indonesia. Nature.

[14] Morriss-Kay G.M. (2010). The Evolution of Human Artistic Creativity. J. Anat..

[15] Guilford J.P. (1967). Creativity: Yesterday, Today and Tomorrow. J. Creat. Behav..

[16] Mednick S. (1962). The Associative Basis of the Creative Process. Psychol. Rev..

[17] Abraham A. (2018). The Neuroscience of Creativity.

[18] Gabora L. (2016). Honing Theory: A Complex Systems Framework for Creativity. arXiv.

[19] Beaty R.E., Seli P., Schacter D.L. (2019). Network Neuroscience of Creative Cognition: Mapping Cognitive Mechanisms and Individual Differences in the Creative Brain. Curr. Opin. Behav. Sci..

[20] Jung R.E. (2013). The Structure of Creative Cognition in the Human Brain. Front. Hum. Neurosci..

[21] Ellamil M., Dobson C., Beeman M., Christoff K. (2012). Evaluative and Generative Modes of Thought during the Creative Process. NeuroImage.

[22] Saggar M., Quintin E.-M., Kienitz E., Bott N.T., Sun Z., Hong W.-C., Chien Y., Liu N., Dougherty R.F., Royalty A. (2015). Pictionary-Based FMRI Paradigm to Study the Neural Correlates of Spontaneous Improvisation and Figural Creativity. Sci. Rep..

[23] De Pisapia N., Bacci F., Parrott D., Melcher D. (2016). Brain Networks for Visual Creativity: A Functional Connectivity Study of Planning a Visual Artwork. Sci. Rep..

[24] Dietrich A. (2019). Where in the Brain Is Creativity: A Brief Account of a Wild-Goose Chase. Curr. Opin. Behav. Sci..

[25] Chamberlain R., McManus I.C., Brunswick N., Rankin Q., Riley H., Kanai R. (2014). Drawing on the Right Side of the Brain: A Voxel-Based Morphometry Analysis of Observational Drawing. NeuroImage.

[26] Shi B., Cao X., Chen Q., Zhuang K., Qiu J. (2017). Different Brain Structures Associated with Artistic and Scientific Creativity: A Voxel-Based Morphometry Study. Sci. Rep..

[27] Schlegel A., Alexander P., Fogelson S.V., Li X., Lu Z., Kohler P.J., Riley E., Peter U.T., Meng M. (2015). The Artist Emerges: Visual Art Learning Alters Neural Structure and Function. NeuroImage.

[28] Xurui T., Yaxu Y., Qiangqiang L., Yu M., Bin Z., Xueming B. (2018). Mechanisms of Creativity Differences between Art and Non-Art Majors: A Voxel-Based Morphometry Study. Front. Psychol..

[29] Sporns O. (2014). Contributions and Challenges for Network Models in Cognitive Neuroscience. Nat. Neurosci..

[30] Kowatari Y., Lee S.H., Yamamura H., Nagamori Y., Levy P., Yamane S., Yamamoto M. (2009). Neural Networks Involved in Artistic Creativity. Hum. Brain Mapp..

[31] Seeley W.W., Menon V., Schatzberg A.F., Keller J., Glover G.H., Kenna H., Reiss A.L., Greicius M.D. (2007). Dissociable Intrinsic Connectivity Networks for Salience Processing and Executive Control. J. Neurosci..

[32] De Pisapia N., Repovs G., Braver T.S., Sun R. (2008). Computational Models of Attention and Cognitive Control.

[33] Kenett Y.N., Medaglia J.D., Beaty R.E., Chen Q., Betzel R.F., Thompson-Schill S.L., Qiu J. (2018). Driving the Brain towards Creativity and Intelligence: A Network Control Theory Analysis. Neuropsychologia.

[34] Chrysikou E.G. (2019). Creativity in and out of (Cognitive) Control. Curr. Opin. Behav. Sci..

[35] Niendam T.A., Laird A.R., Ray K.L., Dean Y.M., Glahn D.C., Carter C.S. (2012). Meta-Analytic Evidence for a Superordinate Cognitive Control Network Subserving Diverse Executive Functions. Cogn. Affect. Behav. Neurosci..

[36] Raichle M.E. (2015). The Brain’s Default Mode Network. Annu. Rev. Neurosci..

[37] Zabelina D.L., Andrews-Hanna J.R. (2016). Dynamic Network Interactions Supporting Internally-Oriented Cognition. Curr. Opin. Neurobiol..

[38] Buckner R.L., DiNicola L.M. (2019). The Brain’s Default Network: Updated Anatomy, Physiology and Evolving Insights. Nat. Rev. Neurosci..

[39] Park H.R., Kirk I.J., Waldie K.E. (2015). Neural Correlates of Creative Thinking and Schizotypy. Neuropsychologia.

[40] Madore K.P., Thakral P.P., Beaty R.E., Addis D.R., Schacter D.L. (2019). Neural Mechanisms of Episodic Retrieval Support Divergent Creative Thinking. Cereb. Cortex.

[41] Chen Q., Beaty R.E., Qiu J. (2020). Mapping the Artistic Brain: Common and Distinct Neural Activations Associated with Musical, Drawing, and Literary Creativity. Hum. Brain Mapp..

[42] Stevenson C., Baas M., van der Maas H. (2021). A Minimal Theory of Creative Ability. J. Intell..

[43] Pascual-Leone A., Amedi A., Fregni F., Merabet L.B. (2005). The Plastic Human Brain Cortex. Annu. Rev. Neurosci..

[44] Medaglia J.D., Huang W., Karuza E.A., Kelkar A., Thompson-Schill S.L., Ribeiro A., Bassett D.S. (2018). Functional Alignment with Anatomical Networks Is Associated with Cognitive Flexibility. Nat. Hum. Behav..

[45] Gu S., Pasqualetti F., Cieslak M., Telesford Q.K., Alfred B.Y., Kahn A.E., Medaglia J.D., Vettel J.M., Miller M.B., Grafton S.T. (2015). Controllability of Structural Brain Networks. Nat. Commun..

[46] Hermundstad A.M., Bassett D.S., Brown K.S., Aminoff E.M., Clewett D., Freeman S., Frithsen A., Johnson A., Tipper C.M., Miller M.B. (2013). Structural Foundations of Resting-State and Task-Based Functional Connectivity in the Human Brain. Proc. Natl. Acad. Sci. USA.

[47] Woollett K., Spiers H.J., Maguire E.A. (2009). Talent in the Taxi: A Model System for Exploring Expertise. Philos. Trans. R. Soc. Lond. B Biol. Sci..

[48] Gobet F. (2013). Expertise vs. Talent. Talent. Dev. Excell..

[49] Goel V., Grafman J. (2000). Role of the Right Prefrontal Cortex in Ill-Structured Planning. Cogn. Neuropsychol..

[50] Miller B.L., Hou C.E. (2004). Portraits of Artists: Emergence of Visual Creativity in Dementia. Arch. Neurol..

[51] Drago V., Foster P.S., Okun M.S., Haq I., Sudhyadhom A., Skidmore F.M., Heilman K.M. (2009). Artistic Creativity and DBS: A Case Report. J. Neurol. Sci..

[52] Rankin K.P., Liu A.A., Howard S., Slama H., Hou C.E., Shuster K., Miller B.L. (2007). A Case-Controlled Study of Altered Visual Art Production in Alzheimer’s and FTLD. Cogn. Behav. Neurol..

[53] Sunavsky A., Poppenk J. (2020). Neuroimaging Predictors of Creativity in Healthy Adults. NeuroImage.

[54] Chrysikou E.G., Wertz C., Yaden D.B., Kaufman S.B., Bacon D., Wintering N.A., Jung R.E., Newberg A.B. (2020). Differences in Brain Morphometry Associated with Creative Performance in High- and Average-Creative Achievers. NeuroImage.

[55] Wertz C.J., Chohan M.O., Flores R.A., Jung R.E. (2020). Neuroanatomy of Creative Achievement. NeuroImage.

[56] Norman K.A., Polyn S.M., Detre G.J., Haxby J.V. (2006). Beyond Mind-Reading: Multi-Voxel Pattern Analysis of FMRI Data. Trends Cogn. Sci..

[57] Grecucci A., Dadomo H., Salvato G., Lapomarda G., Sorella S., Messina I. Two Grey–White Matter Circuits Separate Borderline Personality Disorder from Controls and Mediate the Relationship between Specific Childhood Traumas and Symptoms. A mCCA+ jICA and Random Forest Approach. (Under Review, Preprint Available). https://www.preprints.org/manuscript/202302.0089/v1.

[58] Ghomroudi P.A., Scaltritti M., Grecucci A. (2023). Decoding reappraisal and suppression from neural circuits: A combined supervised and unsupervised machine learning approach. Cogn. Affect. Behav. Neurosci..

[59] Grecucci A., Lapomarda G., Messina I., Monachesi B., Sorella S., Siugzdaite R. (2022). Structural Features Related to Affective Instability Correctly Classify Patients with Borderline Personality Disorder. A Supervised Machine Learning Approach. Front. Psychiatry.

[60] Caria A., Grecucci A. (2022). Neuroanatomical Predictors of Real-Time FMRI-Based Emotional Brain Regulation. Psychophys.

[61] Rondina J.M., Ferreira L.K., de Souza Duran F.L., Kubo R., Ono C.R., Leite C.C., Smid J., Nitrini R., Buchpiguel C.A., Busatto G.F. (2018). Selecting the Most Relevant Brain Regions to Discriminate Alzheimer’s Disease Patients from Healthy Controls Using Multiple Kernel Learning: A Comparison across Functional and Structural Imaging Modalities and Atlases. NeuroImage Clin..

[62] Mourao-Miranda J., Reinders A.A.T.S., Rocha-Rego V., Lappin J., Rondina J., Morgan C., Morgan K.D., Fearon P., Jones P.B., Doody G.A. (2012). Individualized Prediction of Illness Course at the First Psychotic Episode: A Support Vector Machine MRI Study. Psychol. Med..

[63] Melcher D., Bacci F. (2013). Perception of Emotion in Abstract Artworks: A Multidisciplinary Approach. Prog. Brain Res..

[64] Glazek K. (2012). Visual and Motor Processing in Visual Artists: Implications for Cognitive and Neural Mechanisms. Psychol. Aesthet. Creat. Arts.

[65] Zabicki A., de Haas B., Zentgraf K., Stark R., Munzert J., Krüger B. (2019). Subjective Vividness of Motor Imagery Has a Neural Signature in Human Premotor and Parietal Cortex. Neuroimage.

[66] Bacci F. (2013). Resonance: Snapshots of Creativity in the Brain, exh.cat.

[67] Penny W., Friston K., Ashburner J., Kiebel S., Nichols T. (2006). Statistical Parametric Mapping: The Analysis of Functional Brain Images.

[68] Gaser C., Dahnke R., Thompson P.M., Kurth F., Luders E., Initiative A.D.N. (2022). CAT—A Computational Anatomy Toolbox for the Analysis of Structural MRI Data. BioRxiv.

[69] Ashburner J. (2007). A Fast Diffeomorphic Image Registration Algorithm. Neuroimage.

[70] Orsenigo C., Vercellis C. (2012). Kernel Ridge Regression for out-of-Sample Mapping in Supervised Manifold Learning. Expert Syst. Appl..

[71] Dadomo H., Salvato G., Lapomarda G., Ciftci Z., Messina I., Grecucci A. (2022). Structural Features Predict Sexual Trauma and Interpersonal Problems in Borderline Personality Disorder but Not in Controls: A Multi-Voxel Pattern Analysis. Front. Hum. Neurosci..

[72] Schrouff J., Rosa M.J., Rondina J.M., Marquand A.F., Chu C., Ashburner J., Phillips C., Richiardi J., Mourão-Miranda J. (2013). PRoNTo: Pattern Recognition for Neuroimaging Toolbox. Neuroinformatics.

[73] Beaty R.E., Benedek M., Barry Kaufman S., Silvia P.J. (2015). Default and Executive Network Coupling Supports Creative Idea Production. Sci. Rep..

[74] Tzourio-Mazoyer N., Landeau B., Papathanassiou D., Crivello F., Etard O., Delcroix N., Mazoyer B., Joliot M. (2002). Automated Anatomical Labeling of Activations in SPM Using a Macroscopic Anatomical Parcellation of the MNI MRI Single-Subject Brain. Neuroimage.

[75] Shen W., Yuan Y., Liu C., Luo J. (2017). The Roles of the Temporal Lobe in Creative Insight: An Integrated Review. Think. Reason..

[76] Jung R.E., Segall J.M., Jeremy Bockholt H., Flores R.A., Smith S.M., Chavez R.S., Haier R.J. (2010). Neuroanatomy of Creativity. Hum. Brain Mapp..

[77] Sawyer R.K. (2011). Explaining Creativity: The Science of Human Innovation.

[78] Li W., Li G., Ji B., Zhang Q., Qiu J. (2019). Neuroanatomical Correlates of Creativity: Evidence from Voxel-Based Morphometry. Front. Psychol..

[79] Huang P., Qiu L., Shen L., Zhang Y., Song Z., Qi Z., Gong Q., Xie P. (2013). Evidence for a Left-over-right Inhibitory Mechanism during Figural Creative Thinking in Healthy Nonartists. Hum. Brain Mapp..

[80] Jung R.E., Flores R.A., Hunter D. (2016). A New Measure of Imagination Ability: Anatomical Brain Imaging Correlates. Front. Psychol..

[81] Squire L.R., Stark C.E., Clark R.E. (2004). The Medial Temporal Lobe. Annu. Rev. Neurosci..

[82] Beaty R.E., Benedek M., Silvia P.J., Schacter D.L. (2016). Creative Cognition and Brain Network Dynamics. Trends Cogn. Sci..

[83] Jauk E., Neubauer A.C., Dunst B., Fink A., Benedek M. (2015). Gray Matter Correlates of Creative Potential: A Latent Variable Voxel-Based Morphometry Study. Neuroimage.

[84] Benedek M., Schües T., Beaty R.E., Jauk E., Koschutnig K., Fink A., Neubauer A.C. (2018). To Create or to Recall Original Ideas: Brain Processes Associated with the Imagination of Novel Object Uses. Cortex.

[85] Fink A., Grabner R.H., Benedek M., Reishofer G., Hauswirth V., Fally M., Neuper C., Ebner F., Neubauer A.C. (2009). The Creative Brain: Investigation of Brain Activity during Creative Problem Solving by Means of EEG and FMRI. Hum. Brain Mapp..

[86] McPherson M.J., Barrett F.S., Lopez-Gonzalez M., Jiradejvong P., Limb C.J. (2016). Emotional Intent Modulates the Neural Substrates of Creativity: An FMRI Study of Emotionally Targeted Improvisation in Jazz Musicians. Sci. Rep..

[87] Bashwiner D.M., Wertz C.J., Flores R.A., Jung R.E. (2016). Musical Creativity “Revealed” in Brain Structure: Interplay between Motor, Default Mode and Limbic Networks. Sci. Rep..

[88] Wolff M., Vann S.D. (2019). The Cognitive Thalamus as a Gateway to Mental Representations. J. Neurosci..

[89] Szameitat A.J., McNamara A., Shen S., Sterr A. (2012). Neural Activation and Functional Connectivity during Motor Imagery of Bimanual Everyday Actions. PLoS ONE.

[90] Takeuchi H., Taki Y., Nouchi R., Yokoyama R., Kotozaki Y., Nakagawa S., Sekiguchi A., Iizuka K., Hanawa S., Araki T. (2020). Originality of Divergent Thinking Is Associated with Working Memory–Related Brain Activity: Evidence from a Large Sample Study. NeuroImage.

[91] Pinho A.L., de Manzano O., Fransson P., Eriksson H., Ullen F. (2014). Connecting to Create: Expertise in Musical Improvisation Is Associated with Increased Functional Connectivity between Premotor and Prefrontal Areas. J. Neurosci..

[92] Brain Networks Underlying Mental Imagery of Auditory and Visual Information—Zvyagintsev—2013—European Journal of Neuroscience—Wiley Online Library. https://onlinelibrary.wiley.com/doi/full/10.1111/ejn.12140.

[93] Chen X., Liang S., Pu W., Song Y., Mwansisya T.E., Yang Q., Liu H., Liu Z., Shan B., Xue Z. (2015). Reduced Cortical Thickness in Right Heschl’s Gyrus Associated with Auditory Verbal Hallucinations Severity in First-Episode Schizophrenia. BMC Psychiatry.

[94] Pearson J. (2019). The Human Imagination: The Cognitive Neuroscience of Visual Mental Imagery. Nature Reviews. Neuroscience.

[95] Winlove C.I.P., Milton F., Ranson J., Fulford J., MacKisack M., Macpherson F., Zeman A. (2018). The Neural Correlates of Visual Imagery: A Co-Ordinate-Based Meta-Analysis. Cortex.

[96] Pidgeon L.M., Grealy M., Duffy A.H.B., Hay L., McTeague C., Vuletic T., Coyle D., Gilbert S.J. (2016). Functional Neuroimaging of Visual Creativity: A Systematic Review and Meta-analysis. Brain Behav..

[97] Solso R.L. (2001). Brain Activities in a Skilled versus a Novice Artist: An FMRI Study. Leonardo.

[98] Varoquaux G. (2018). Cross-Validation Failure: Small Sample Sizes Lead to Large Error Bars. Neuroimage.

[99] Vabalas A., Gowen E., Poliakoff E., Casson A.J. (2019). Machine Learning Algorithm Validation with a Limited Sample Size. PLoS ONE.

[100] Turner B.O., Santander T., Paul E.J., Barbey A.K., Miller M.B. (2019). Reply to: FMRI Replicability Depends upon Sufficient Individual-Level Data. Commun. Biol..

[101] Morrison R.G., Wallace B. (2001). Imagery Vividness, Creativity and the Visual Arts. J. Ment. Imag..

[102] Haynes J.-D., Rees G. (2006). Decoding Mental States from Brain Activity in Humans. Nat. Rev. Neurosci..

